# Intimate Partner Violence and Structural Violence in the Lives of Incarcerated Women: A Mixed-Method Study in Rural New Mexico

**DOI:** 10.3390/ijerph18126185

**Published:** 2021-06-08

**Authors:** Shilo St. Cyr, Elise Trott Jaramillo, Laura Garrison, Lorraine Halinka Malcoe, Stephen R. Shamblen, Cathleen E. Willging

**Affiliations:** 1 Sexual Violence Prevention and Response Office, University of British Columbia, Kelowna, BC V1V 1V7, Canada; shilo.stcyr@ubc.ca; 2 Pacific Institute for Research and Evaluation, Albuquerque, NM 87106, USA; laura.garrison53@gmail.com (L.G.); cwillging@pire.org (C.E.W.); 3 Joseph J. Zilber School of Public Health, University of Wisconsin–Milwaukee, Milwaukee, WI 53205, USA; malcoe@uwm.edu; 4 Pacific Institute for Research and Evaluation, Louisville, KY 40202, USA; sshamblen@pire.org

**Keywords:** intimate partner violence, rurality, structural violence, incarceration, race/ethnicity, substance use

## Abstract

Intimate partner violence (IPV) is a common feature in the lives of incarcerated women returning to rural communities, enhancing their risk of mental ill-health, substance use, and recidivism. Women’s experiences of IPV intersect with challenges across multiple social–ecological levels, including risky or criminalizing interpersonal relationships, geographic isolation, and persistent gender, racial, and economic inequities. We conducted quantitative surveys and qualitative interviews with 99 incarcerated women in New Mexico who were scheduled to return to micropolitan or non-core areas within 6 months. Quantitative and qualitative data were analyzed separately and then triangulated to identify convergences and divergences in data. The findings underscore how individual and interpersonal experiences of IPV, substance use, and psychological distress intersect with broad social inequities, such as poverty, lack of supportive resources, and reluctance to seek help due to experiences of discrimination. These results point to the need for a more proactive response to the mutually constitutive cycle of IPV, mental distress, incarceration, and structures of violence to improve reentry for women returning to rural communities. Policy and treatment must prioritize socioeconomic marginalization and expand community resources with attention to the needs of rural women of color.

## 1. Introduction

Cynthia is a 26-year-old Native American woman from a rural community in New Mexico, serving her second prison sentence for violating parole conditions by drinking with her boyfriend in a residential treatment facility. She will leave prison in six months and plans to live with him. She is fearful of their reunion, as her boyfriend drinks heavily and has physically and sexually abused her. Cynthia has no safe place to go upon release from prison. She does not feel safe near her male relatives and remains traumatized after witnessing her stepfather beat up her brother. She has been suicidal at times. As a teenager, Cynthia stopped going to school, repeatedly ran away from home, and drank alcohol to cope. Now, she expects that her criminal record and lack of a high school diploma will prevent her from getting a job and providing for her children.

Intimate partner violence (IPV) is a prominent feature in the pathways that lead women such as Cynthia to prison and enhance their risk of mental ill-health, substance use, and recidivism. In New Mexico, a rural state with a complex history of colonialism (i.e., political occupation and economic exploitation), women’s experiences of IPV intersect with challenges across multiple social–ecological levels [1]—from individual struggles (e.g., mental distress, substance use), to risky or criminalizing interpersonal relationships, to community characteristics that make it difficult for them to get help (e.g., geographic isolation), all of which are compounded by persistent gender, racial, and economic inequities [2,3]. Given these substantial and intersecting challenges, there is a continued need to understand and plan for the complex correctional and reentry needs of women in these circumstances. Using a mixed-method approach, we examine women prisoners’ experiences of IPV as one facet of the social–ecological context that shapes their perspectives on their post-prison lives and limits what is possible for them upon release.

### Intimate Partner Violence, Rurality, and Incarceration of Women

Although precise definitions of intimate partner violence (IPV) differ, IPV generally refers to violence between people in a current and/or past spousal or dating relationship for longer than one month, including physical and sexual violence, threats, and emotional and financial abuse [4]. Severe and chronic IPV is common among incarcerated women [5], and is correlated with mental distress and substance use [6,7]. 

Rurality is an additional factor that increases women’s exposure to IPV, rape, and homicide [8,9]. “Micropolitan” (≤50,000 persons) and “non-core” (≤10,000 persons) regions [10] are characterized by denser social networks, reduced autonomy, and greater vulnerability to economic and social changes compared to metropolitan centers [11,12]. In part due to these factors, rural women experience more poverty and have reduced access to IPV intervention resources compared to rural men and urban residents, as well as increased risk of mental and substance-related issues [13,14]. Stigma and loss of confidentiality prevents many rural residents from seeking social and health services [15,16]. These factors complicate women’s abilities to escape violent relationships [17]. 

Feminist pathways theory indicates that physical and sexual violence throughout the life course and especially in childhood are common to multiple causal pathways that lead women to mental distress, substance use, criminalized behaviors, and incarceration [18,19,20]. Moreover, IPV continues to affect women’s lives after reentry and is connected to their likelihood of recidivism [19,21]. Experiences of IPV are pervasive in the lives of incarcerated women returning to rural communities [22,23], largely due to the scarcity of supportive resources, employment opportunities, adequate and affordable housing, and the strong presence of traditional ideologies of power, patriarchy, and privacy that structure gender relationships and policing in rural states [24,25]. Studies also link racism, poverty, and patriarchy with the abuse and incarceration of women, particularly Native Americans [26,27,28]. While IPV and criminality are not inherently linked, research demonstrates that they are strongly related through the constellation of factors that collectively leave rural women more vulnerable to such adverse experiences [29,30] and reduce their chances to escape unhealthy and unsafe situations [31]. 

In New Mexico, a history of colonization has dispossessed Hispanic and Native American citizens from land and natural resource rights, contributing to the impoverishment of the state’s many rural and non-White regions. Structures of racism contribute to persistent exclusions, including discrimination and disparate access to housing, education, employment, and health care. Such disparities lead to social and psychological suffering (e.g., poverty, mental illness, and substance use issues). In New Mexico and elsewhere, a history of patriarchy and its ongoing effects have been implicated in the systematic devaluation of, and violence against, women—especially women of color [23,27,32]. In previous studies, we showed that women leaving prison in this environment must rely on informal and insecure networks of family and friends—many of whom also struggle with substance abuse—for financial assistance, housing, and transportation. They experience stigmatization and have trouble avoiding unhealthy relationships and forming supportive bonds in situations of social intimacy and isolation [3]. Women without family often return to abusive partners or attempt to find transitional housing or short-term treatment facilities, most of which are far from their homes [22]. These challenges are compounded by racial and gender discrimination. Criminalized women also encounter negative gendered ideologies from law enforcement, healthcare providers, and others who view them as inherently criminal or beyond help [2,9,10]. In this study, we examine women’s IPV experiences in relation to the racial, socioeconomic, and health inequities that are common to rural New Mexico and that shape women’s perspectives on their post-incarceration life. 

## 2. Materials and Methods

We implemented a mixed-method research design in which qualitative and quantitative data were collected and analyzed simultaneously to determine convergence and/or divergence points. This design allowed us to examine both the content of women’s demographic/socioeconomic characteristics and substance use, mental health, and IPV histories (quantitative) and the context of their perspectives and experiences (qualitative) [33]. We asked: how have women prisoners returning to rural communities experienced IPV prior to incarceration? How have experiences of IPV intersected with inequities at other social-ecological levels, including mental health and substance use, socioeconomic inequality, and racial/ethnic discrimination? How do these experiences impact their prospects for successful reentry? We hypothesized that racial disparities (measured by race/ethnicity) and socioeconomic marginalization (measured as economic hardship, low education, and high unemployment) would correlate with increased vulnerability to psychological (measured as substance use and mental distress) and physiological (measured as childhood abuse and IPV) harms for incarcerated women returning to rural areas. Our analysis foregrounds the voices of women in order to descriptively account for the specific ways in which experiences of IPV related to other social and health inequities and influence women’s senses of their own capacities to attain supportive relationships, education, safe housing, and lives that are free from harm. 

### 2.1. Setting and Procedures

In New Mexico, Hispanics and Native Americans comprise over 60% of 2,096,829 residents, nearly 700,000 of whom live in rural areas [34]. Of its 33 counties, 26 are rural [35]. Poverty is pervasive in these areas as they exhibit lags in population and job growth [36], as well as lower educational attainment and more housing stress than urban counties [37]. Many are medically underserved, meaning that there is a shortage of primary care services [38]. Historically, New Mexico has suffered from among the highest rates of substance-related illness and suicide nationwide, disproportionately affecting people of color [39]. 

As part of a broader study of women’s reentry needs in rural communities, we recruited participants between March and August 2009 from the state’s only women’s prison. All general population inmates scheduled to return to micropolitan or non-core areas within 6 months were eligible. Each candidate was invited to participate in one structured survey and one semi-structured interview. Exclusion criteria determined by the prison warden were active or recent suicidality, the diagnosis of a chronic mental health condition with functional impairment, or determination as a security risk. Of the 103 women invited to participate, two met the exclusion criteria, one declined, and 99 agreed to participate. The Institutional Review Board of the Pacific Institute for Research and Evaluation approved the study design, instruments, and written informed consent protocols.

### 2.2. Quantitative Survey

To assess social and health inequities, the 60- to 90-min survey included closed-ended questions on demographic and socioeconomic status, drug and alcohol use, mental health, trauma history, and recent IPV. Women reported race/ethnicity, age, sexual orientation, previous incarcerations, and if they were mothers, as well as education level, housing situation at the time they entered prison, and major income source and economic hardship in the 6 months before prison. Information on urban vs. rural background prior to incarceration was not collected systematically, although our qualitative data affirms that many women had mixed backgrounds, with some migrating between cities and rural areas. Substance use disorder (SUD) was assessed using the first nine questions of the Texas Christian University Drug Screen II [40]. Current mental distress was determined using the MINI International Neuropsychiatric Interview [41] to assess Axis I conditions, including major depression, mania/hypomania, panic disorder, post-traumatic stress disorder (PTSD), psychotic disorders, mood disorder with psychotic features, and generalized anxiety disorder [42]. 

We assessed histories of childhood sexual and physical abuse using the Trauma History Questionnaire [43]. Women were asked to report both sexual and physical trauma and the number of times and the ages for each trauma type they experienced. We characterized any physical or sexual trauma before the age of 18 as child abuse. Finally, we assessed recent IPV via the 30-item Composite Abuse Scale (CAS) with a modified timeframe referring to the 12-month period preceding imprisonment [4]. Women reported the frequency of different types of abuse (described below) from 0–5 (never, once, several times, once/month, once/week, daily) experienced during an adult intimate relationship, defined as a husband, partner, or boy/girlfriend for longer than a month. The 30 CAS items for total IPV (IPV-T) were subdivided into severe combined IPV (IPV-SC) containing 8 items; emotional IPV (IPV-E) containing 11 items; physical IPV (IPV-P) containing 7 items; and harassment IPV (IPV-H) containing 4 items (Hegarty et al., 2005; see Table 1). Cut off scores of 7 for IPV-T, 1 for IPV-SC, 3 for IPV-E, 2 for IPV-P, and 2 for IPV-H were determined based on the CAS Manual [44].

### 2.3. Quantitative Analysis

The structured interview data were entered into an Access database, validated, and analyzed with SPSS, Version 19 [45]. To assess the prior risk factors that were associated with experiences of IPV, the outcome variable for quantitative analyses was IPV (specifically, IPV-T, IPV-SC, IPV-E, IPV-P, and IPV-H). The IPV outcome variable was a binary variable as determined by the cut-off scores. 

We calculated odds ratios (ORs) to estimate univariate associations of IPV (IPV-T, IPV-E, IPV-P, and IPV-H) with socioeconomic variables and the measures of childhood abuse, SUD, and mental distress. To analyze the associations without increasing Type 1 error, we conducted a series of multivariate logistic regression models, one for each IPV type. For each model, we first entered all variables associated with each IPV measure at *p* < 0.10 in univariate analyses, controlling for age, since it is a known determinant of IPV [46]. We then deleted variables that were not significant at *p* < 0.05 to arrive at the final models.

### 2.4. Qualitative Interviews and Analysis

Qualitative interviews of 90- to 190-min included open-ended questions on participant backgrounds, home life and personal relationships, social support, physical and mental health, substance use, prior incarceration(s), preparation for reentry, and perceptions of community resources. The digitally recorded interviews were transcribed into an electronic database and analyzed through a series of iterative readings using the software NVivo [47]. We developed a descriptive coding scheme from transcripts based on the interview questions. We then engaged in *open* and *focused coding* to identify new themes and determine which themes were repeated often or represented unusual or particular concerns. We grouped together themes related to key sensitizing concepts from the literature on IPV and other risk factors (i.e., socioeconomic marginalization, racism, and gender inequity). These concepts provided “a general sense of reference” [48] and supplied descriptive data based on the words of participants, enabling us to examine their relevance and meaning. We created detailed memos that described and linked codes to each theme and collectively reviewed the findings. We grouped codes with similar content or meaning into broad themes linked to larger segments of women’s narratives [49,50].

### 2.5. Triangulation

Triangulation involves summarizing and identifying convergences and divergences in data (quantitative and qualitative) and then integrating results to create a holistic picture of IPV experiences and post-incarceration prospects among women in this study. First, we examined quantitative and qualitative results separately. We then created a matrix for the side-by-side comparison of each data set related to childhood abuse, mental health and substance use, socioeconomic conditions, and race and ethnicity (Table 5). We examined: (1) *convergence* (i.e., whether results provide the same answer to questions); (2) *expansion* (i.e., whether unanticipated findings of one data set can be explained by findings in the other), and (3) *complementarity* (i.e., whether qualitative results can contextualize quantitative results) [33]. 

## 3. Results

### 3.1. Quantitative Results

The 99 women in the study were 20 to 56 years old (M = 35.2, SD = 8.4) and identified as Hispanic (*n* = 33), Native American (*n* = 33), non-Hispanic White (*n* = 32), and African American (*n* = 1). Seventy-nine percent identified as heterosexual, 6% as gay/lesbian, and 15% as bisexual. Most (89%) women identified as mothers (Table 2). Forty-eight percent had been incarcerated in the state prison more than once, 41% before they turned 18. At the time of their interviews, the women had been incarcerated for between 9 to 36 months. About 50% of women had attained a high school degree or equivalent. In the 6 months before incarceration, a third (33%) were employed, 47% received welfare and/or food stamps, 56% did not live in their own residence, and over one-half (51%) had worried about not having enough income to meet basic needs, including food and housing (Table 3). 

Current mental distress and SUD were pronounced (89%). Alcohol (37%) and methamphetamine (38%) use prevailed. Among women with Axis 1 mental health issues (*n* = 50), mood (50%) and anxiety (68%) disorders, particularly PTSD (26%), were most common. Sixty-one percent reported child physical and/or sexual abuse with an average age of onset of 8 years. Ninety-one percent reported some IPV in the 12 months prior to incarceration. With a cut-off of 3, 75% were positive for IPV-T; 51% reported IPV-SC; 64% were positive for IPV-E with the cut off of 3; 57% were positive for IPV-P with the cut off of 2, and 50% were positive for IPV-H with the cut off of 2. 

In univariate analyses (Table 3), IPV-T, IPV-SC, IPV-E, and IPV-H were significantly associated with age; 80.6% of women under age 30 reported IPV-T compared with 83.8% of women aged 30−39 and 58.1% of women aged 40 and older. Housing was significantly associated with IPV-P and IPV-H. Women living in their own housing at the time of incarceration had increased prevalence of IPV-P and IPV-H compared to women who were homeless or living in someone else’s home. Although not significant at an alpha of 0.05, there was a 2.2-fold increased likelihood of IPV-SC among women who had their own housing compared to women who did not, likely due to a lack of housing options away from abusive partners (see Section 3.3). Women with a history of child abuse had increased prevalence of IPV-T compared to women who did not. Race/ethnicity, economic hardship, and income source were not significantly associated with any IPV measure. Women with at least one mental health issue had significantly increased odds of all IPV types compared to women who did not have mental health issues. Women who reported illegal substance use had increased prevalence of IPV-H. Mental distress with SUD and SUD alone were not significantly associated with any IPV measure.

Table 4 presents final logistic regression models for four IPV measures (excluding IPV-E, as only age was significant in the univariate model). In the IPV-T model, child abuse did not remain significant when controlling for age. In the IPV-P model, housing remained significant when controlling for age. The IPV-SC and IPV-H models were alike; in addition to age, they contained two variables—housing and mental distress—with similar effect sizes. There was a 2.60-fold adjusted association of housing with IPV-H and a 3.09-fold adjusted association between mental distress and IPV-H. However, the choice of drugs dropped out of the IPV-H model. 

### 3.2. Qualitative Results

Interviews with women revealed common and interconnected experiences of abuse, struggles with mental health and substance use, poverty, and discrimination, which both compounded their vulnerability to IPV and limited their options upon returning home from prison.

#### 3.2.1. Experiences of Abuse

Participants’ life histories commonly included stories of abuse in childhood that continued into adulthood. As our quantitative results confirm, multiple women reported being raped or sexually assaulted by a relative during childhood. Their experiences of childhood abuse were often complicated by feelings of confusion and guilt instilled by secrecy or lack of support from other relatives. One woman who was sexually molested by a cousin as a child, explained that she had felt unsupported by her family during this trauma, ultimately coming to feel personally responsible for the abuse she endured. Echoing the stories of others, this woman reported that she had developed substance dependence after family members and community counseling services failed to address her requests for assistance. The majority of women reflected on the connections between childhood abuse and patterns of lifelong violence. One woman stated, “My dad was very emotionally and physically abusive with my entire family and I went from that to my husband, who was the master of emotional abuse. And then I went from him to my ex-boyfriend, who was physically and emotionally [abusive].” 

Fear of abuse was a prominent theme in women’s outlooks on their post-prison lives. Many said they were fearful of present or ex-partners, explaining, for example, “I don’t want to have anything to do with him. But he does know where my parents live, and he knows I was living there.” A second woman reported that she had heard that an abusive ex-boyfriend was inquiring about her whereabouts. When asked about potential threats after their release, other women simply stated, “Just my husband” or “My exes.” A third woman explained, “Sometimes I do [feel fear]. He wrote me a letter and told me that ‘No matter what, you’re from the *barrio*. We’ll see each other again.’” A fourth resolved to end her relationship with an ex-boyfriend, commenting, “I’m gonna have to tell him face-to-face … that we can’t do this anymore because one of these days … I’m afraid that I’m gonna die [at] his hands.” A fifth considered whether she might need a restraining order against a former partner.

#### 3.2.2. Mental Health and Substance Use

Fear of IPV reportedly impacted women’s mental health and use of alcohol and drugs. When asked about the impact of abuse in her life, one woman responded, “The nightmares and panic attacks and voices. I’m nervous all the time. I’m always looking over my shoulder.” Another woman linked abuse experiences to her current mental health: “Maybe that’s why I am the way I am right now, like with the depression. [The abuse] really affected me bad.” Some women admitted to self-medicating with alcohol and drugs to cope with these feelings. Echoing the experiences of her fellow inmates who had been in abusive relationships, a third woman explained, “Alcohol made me feel good. It made things go away.”

Substance use played an influential role in women’s decisions to remain in abusive relationships. One woman talked about enduring abuse because her partner provided a steady supply of drugs: “Life was hopeless. Everything was hopeless, so I just stayed with him and he was my drug provider. He always brought the drugs home. He was getting me high every single day.” When the women contemplated their post-prison lives, many expressed fear for their mental health and ability to stay away from substances. For example, one woman commented, “[T]here’s always that fear of me going back to drugs, ‘cause it’s just something that I’ll never know.”

#### 3.2.3. Socioeconomic Conditions

Many women sought out or remained in relationships to fulfill basic needs. One woman stated, “When I was in it, it was hard to get out because I felt like I couldn’t do it by myself.” Difficulty finding alternative housing away from their partners was a major obstacle to women wanting to flee abusive relationships. A second woman stated, “He would hit me. By then, I was already out of my mom’s so it was already too late. And my son took my room that I was living in, so I just stayed with him and thought it was hopeless.” Many women lacked close connection to kin. Estranged from her family, a third woman had relied on the parents of her abusive ex-husband for assistance. 

Women identified IPV, drugs, pregnancy, and school policies as barriers to pursuing education. One woman stated, “I ended up leaving home for him. And that’s the reason why I did not finish high school, because he was jealous.” A second woman explained that drug use contributed to her leaving school, “Before I used to be, like smarter. But now my brain don’t think. And then after that [drug use], I just dropped out.” Pregnancy also prevented multiple women from completing school. A third woman explained, “I got pregnant. I was going to school [and] eventually [I] ended up dropping [out].” Finally, school disciplinary protocols hindered several women from completing their education, especially when their infractions were related to fighting.

Poverty and lack of housing also influenced women’s deliberations about returning to partners after their release from prison. A fourth woman stated, “Let me save up some money so when I get out, I don’t have to go to my boyfriend’s house.” Yet a fifth woman feared for her safety as she expected to be paroled to the home of her abusive ex-husband.

#### 3.2.4. Discrimination

When asked whether they had experienced any discrimination or racism in their lives, women of color reported facing institutional racism in criminal justice, financial, occupational, and educational systems. Participants spoke about profiling by law enforcement, commenting that it was common for rural police officers to pull over vehicles of people of color without due cause. Outside prison, women experienced racism on a daily basis—in banks, schools, and the workforce. “Even going to get a loan for a car. They’re more likely to give a loan to a non-Native than they are to a Native,” said one woman. A second woman stated, “I did not get the job because I was kind of an outcast for being Mexican.” A third woman summed up the quotidian nature of racism: “It makes me mad, but I’ve just learned to live with it, being that I grew up in that border town.”

### 3.3. Mixed-Method Results

Both quantitative and qualitative findings demonstrate the utter pervasiveness of IPV, as well as childhood abuse, mental health and substance use issues, poverty, experiences of racial discrimination, and fear in the lives of incarcerated women, as illustrated in Table 5. The limited variability in the quantitative findings underscores the ubiquity of these experiences, while the qualitative findings provide additional context, meaning, and clarification, shedding light on how IPV intersected with other social and health inequities to increase their continued exposure to harms after release from prison and limit their future life chances.

## 4. Discussion

The women in this study reported high rates of recent IPV (91%), which is consistent with rates among incarcerated women in other states [5]. Our study offers further evidence that, despite variability in age, race, and background, most women experience violence prior to incarceration. Women’s concerns about their post-prison lives are substantiated by research indicating that incarceration escalates the risk of abuse by increasing associated risk factors (i.e., poverty, poor mental health, weak social support systems) [51]. 

Our findings of child victimization are consistent with the broader literature on adverse childhood experiences in rural communities [52] and among prisoners [53], as well as the effects of child abuse on health and IPV risk in adulthood [54]. This study also confirms the association of IPV with mental health and substance use, while shedding light on the specific circumstances that connect these factors to IPV. Although we cannot draw a causal link between mental distress, SUD, and IPV, our qualitative data points to their mutually constitutive nature. In keeping with other research, women reported that violence affected their mental health through persistent and unpredictable fear, depression, and anxiety [14,55], which often prompted alcohol and drug consumption and possibly the necessity to stay with abusive partners who helped sustain their substance use. While the linkage between IPV, mental health, and substance use is widely acknowledged, our findings underscore the grave impact of IPV on women’s mental health, an effect that is often minimized or neglected. For example, our previous research indicates that IPV experiences are largely ignored in common characterizations of women prisoners as needy and manipulative by correctional staff and treatment providers [2].

We found that women with housing at the time of incarceration had an increased risk of most IPV types compared to women who were homeless or precariously housed. This finding was inconsistent with studies that find IPV correlated with homelessness [56]. However, fleeing abusive relationships can also render women homeless [57]. Our findings resonate with research on resource-scarce rural settings, where a lack of housing and distance to IPV support resources are significant barriers to escaping a violent partner [13,58]. The women in our sample anticipated returning to rural areas where colonization and economic marginalization has resulted in a dearth of safe places (e.g., shelter services, subsidized housing), and social isolation prevents them from gaining distance from their abusers. This is consistent with studies identifying these as barriers to fleeing abusive relationships [59,60].

Although we found no significant quantitative association between race/ethnicity and IPV, this finding underscores the prevalence of IPV among all of the women in our study. Nonetheless, our qualitative data showed that women of color encountered racism in criminal justice, financial, educational, and employment settings, potentially foreclosing opportunities to become financially stable. Numerous studies illustrate that such experiences also contribute to poor mental health and substance use [61]. Moreover, encounters with racial discrimination have been shown to be associated with distrust of healthcare systems, thus decreasing the likelihood that women of color will seek help [62]. Experiences of discrimination are thus likely to be a part of the web of IPV, mental health, substance use, and lack of supportive resources that shaped the life histories and prospects of the women in this study.

Our findings underscore how individual and interpersonal experiences of IPV, substance use, and psychological distress intersect with broad social inequities, such as poverty, lack of supportive resources, and reluctance to seek help due to experiences of discrimination within the context of rural New Mexico. Such interconnected challenges are characteristic of “structural violence,” meaning social arrangements that create unequal distributions of power, placing certain populations at greater risk for harm [63,64]. These social arrangements “are structural because they are embedded in the political and economic organization of our social world; they are *violent* because they cause harm to people” [65]. In New Mexico, structures of racism, sexism, and patriarchy are the legacy of colonialism, with both symbolic and material effects, including rural poverty, disparities in access to resources such as housing and health care, and gender discrimination. Popular and scholarly literatures have often tied these disparities to the place and culture of rural New Mexico, stigmatizing Native American and Hispanic residents as criminal, poor, and trapped within intergenerational patterns of substance abuse and ill-health [66,67]. These attitudes figure prominently in women prisoners’ experiences with law enforcement and service providers [2]. Presumptions of criminality also contributed to a 2013 Medicaid freeze that effectively decimated the behavioral health safety net in rural New Mexico, although the fraud allegations that triggered the freeze turned out to be unfounded [68]. In these ways, such structures contribute to and reinforce the effects of IPV in the individual lives of women like those in this study. Moreover, when they considered their upcoming release from prison, the effects of structural violence often constrained women’s perceptions of potential alternatives to abusive relationships and/or criminalized behaviors. Thus, a more proactive response to the mutually constitutive cycle of IPV, mental distress, incarceration and structures of violence is needed to improve reentry for women returning to rural communities.

Reentry preparation and post-incarceration resources for women leaving prison must recognize the influence of structural forces above individual and interpersonal characteristics on women’s health and reentry outcomes. Emerging frameworks in structural competency emphasize training healthcare providers and corrections personnel to recognize and address structural vulnerabilities through “prescriptions” of social and economic supports and resources (e.g., safe housing, food security, and employment) [69]. Such efforts de-stigmatize the struggles of rural women prisoners by familiarizing providers and corrections personnel with their experiences, while simultaneously emphasizing assistance to find and cultivate support for women *within* their home communities, rather than further denouncing those communities as racist, sexist, and poor. One promising evidence-based model is the Critical Time Intervention (CTI) [70], which marshals social and community support systems to target the effects of structural violence and improve reentry outcomes. The CTI is delivered by specially trained case managers who implement tools such as illness management and recovery, supportive housing, and psychosocial skill building. Importantly, the CTI does not simply target individual behaviors, but promotes collaboration with corrections personnel, treatment providers, and health and social workers to leverage and coordinate the fragmented systems of care that are characteristic of rural areas.

In addition to the CTI, other potential supports for women such as those in our study include *Beyond Violence,* an evidence-based psycho-social trauma-informed curriculum that considers individual, relationship, community, and structural factors and has been shown to decrease mental health symptoms, substance use, and violence for women in prison with histories of IPV and trauma [71]. Referrals for harm reduction and treatment options (e.g., safe injection sites, needle distribution programs, opioid agonist therapy, prescription heroin programs, individualized and group trauma-informed counseling, short-term detoxification facilities, and long-term residential treatment) should occur within primary care settings, the *de facto* safety net in medically underserved areas [72]. Women also need housing support as the first step of effective treatment and behavior change [73] and personalized safety planning for living with or leaving an abusive partner [74]. Reentry planning processes must emphasize gender-responsive approaches that acknowledge the role of IPV in women’s pathways to prison and account for the specific risks women face in reentering their home communities [75]. However, further research is critically needed to adapt these and other approaches for culturally and geographically specific and resource-scarce environments such as rural New Mexico [76]. One exemplary approach is a national movement named *Incite! Women of Color Against Violence* [77], which is creating local community driven health clinics, campaigns, and housing programs to address violence against women of color. In New Mexico, a coalition of community-based organizations have formed an Ending Gender-Based Violence Cohort to promote anti-violence using local and state policy advocacy, as well as the promotion of free, bilingual, and holistic direct services. The Cohort advances a number of specific recommendations to reduce violence against marginalized people—including women—in New Mexico. Prominent among these is ensuring that survivors of gender-based violence have a seat at the table when policy is developed so that it effectively reflects their needs. They also emphasize the need to fund alternatives to incarceration and to remove barriers to shelter, health care, and other services for marginalized people of all kinds [78].

Finally, recognizing the role of structural violence in the cycle of IPV and incarceration also entails increasing social and institutional accountability for the barriers that returning prisoners face [79], such as policies and their underlying ideologies that stigmatize the impact of IPV on women’s mental health, or that limit support for resources to meet their structural needs, including education, housing, and employment. 

### Limitations

The cross-sectional design of this study prevented us from establishing temporal links between incarceration, substance use/mental health, and IPV, limiting our ability to quantitatively delineate casual pathways. The small sample size also limited our multivariate analyses. The statistically significant associations should be cautiously interpreted, as we could not assess and reduce all effects of confounding. A ceiling effect limited our ability to undertake statistical analysis between women with any IPV and women with no IPV. Thus, while our findings demonstrate the commonality of IPV and other social and health risk factors, we could not adequately assess quantitatively whether the specific factors we selected were associated with IPV. Although our qualitative findings shed light on the possible effects of IPV on women’s post-prison lives, as well as the potential role played by experiences of racial/ethnic discrimination, we did not assess these effects quantitatively.

The measures used to assess mental distress and substance use have been widely implemented among different racial/ethnic populations. Yet, they are also based on Western-derived diagnostic criteria and may not adequately capture the lived experience of all participants. 

The data in this study were collected in 2009, which may limit their relevance. However, the problem of resource scarcity for women returning to rural communities in New Mexico is equally—if not more—severe today. For example, the loss of the behavioral health safety net in rural New Mexico presents major challenges to women seeking treatment for mental health and substance use issues [68]. Housing options also remain woefully inadequate for women prisoners facing release. A paucity of transitional facilities and halfway houses for women throughout the state contributes to the persistent problem of “in-house parole,” where prisoners remain in prison past the time they are eligible for release because they have nowhere else to go [80]. Consequently, the need for policy and treatment options for women returning to rural areas is as acute as ever.

## 5. Conclusions

Intimate partner violence and associated social and health inequities are widespread for incarcerated women in rural areas. Our mixed-method analysis contributes to a nuanced understanding of the reproduction of long-term abuse and IPV, mental distress, and substance use within a social–ecological context shaped by structures of violence. Examining these structures is a crucial step in understanding the root causes of inequity. Our findings indicate the need for future policy that redresses social and health inequities associated with IPV through institutional and social change. We emphasize that policy and treatment efforts alike must promote structural competency and cultural adaptation of promising health interventions. Policymakers, healthcare providers, and corrections personnel should prioritize efforts to increase access to housing, employment, and education, in addition to expanding supportive community resources and harm reduction strategies. 

## Figures and Tables

**Table 1:** Definition of IPV types.

**Severe combined IPV (IPV-SC):** Use of severe physical force, forceful unwanted sexual behavior, and physical isolation	
CAS items:	Kept me from medical care Used a knife or gun or other weapon Locked me in the bedroom Put foreign objects in my vagina Refused to let me work outside the home Raped me Tried to rape me Took my wallet and left me stranded
**Emotional IPV (IPV-E):** Use of verbal and psychological dominance, and social isolation	
CAS items:	Told me that I was crazy Tried to convince family, friends and children that I was crazy Became upset if dinner/housework wasn’t done when they thought it should be Told me that I wasn’t good enough Tried to keep me from seeing or talking to my family Told me that I was stupid Tried to turn my family, friends, and children against me Did not let me socialize with my female friends Told me that I was ugly Told me no one would ever want me Blamed me for their violence
**Physical abuse (IPV-P):** Use of physical force	
CAS items:	Shook me Hit or tried to hit me with something Pushed, grabbed or shoved me Kicked me, bit me or hit me with a fist Slapped me Threw me Beat me up
**Harassment (IPV-H):** Unwanted contact that causes fear and concern for safety	
CAS items:	Harassed me over the telephone Harassed me at work Followed me Hung around outside my house

**Table 2:** Demographic information of participants by race/ethnicity.

Characteristics	Hispanic (*n* = 33)	American Indian (*n* = 33)	White, Non-Hispanic (*n* = 32)
**Average age**	33.4	37.0	35.3
**Average years of education**	10.7	10.9	11.4
**Orientation** Lesbian Bisexual	9% 18%	9% 21%	0 3%
**Marital status** Never Married Married/Cohabiting Separated/Divorced Widowed	46% 15% 36% 3%	49% 18% 30% 3%	19% 28% 50% 3%
**Average number of children**	2.4	2.4	3.0
**Employment status prior to prison** Working Full-time Working Part-time Unemployed Homemaker Other	30% 6% 36% 21% 6%	42% 9% 30% 0 18%	44% 13% 28% 16% 0

**Table 3:** Univariate analyses of IPV by type.

Factor	*n* ^a^	% IPV ^b^-T	OR	*p*	% IPV-SC ^c^	OR	*p*	% IPV-E ^d^	OR	*p*	% IPV-P ^e^	OR	*p*	% IPV-H ^f^	OR	*p*
**Age, years**																
40+	31	58.1	1.0	**0.034**	29.0	1.0	**0.015**	45.2	1.0	**0.034**	45.2	1.0	0.113	25.8	1.0	**0.003**
30–39	37	83.8	3.7		62.2	4.0		70.3	2.9		54.1	1.4		54.1	3.4	
20–29	31	80.6	3.0		58.1	3.4		74.2	3.5		71.0	3.0		67.7	6.0	
**Race/ethnicity ^g^**																
White, non-Hispanic	32	78.1	1.0	0.633	53.1	1.1	0.730	65.6	1.0	0.860	53.1	1.0	0.829	56.2	1.4	0.525
American Indian	33	69.7	0.6		45.4	1.0		60.6	0.8		60.6	1.4		42.4	1.0	
Hispanic	33	78.8	1.0		54.5	1.1		66.7	1.0		57.6	1.2		51.5	1.2	
**Child abuse**																
No	39	64.1	1.0	**0.049**	41.0	1.0	0.128	56.4	1.0	0.228	51.2	1.0	0.393	38.5	1.0	0.077
Yes	60	81.7	2.5		56.7	1.9		68.3	1.7		60.0	1.4		56.7	1.4	
**Primary income source**																
Other	52	75.8	1.2	0.744	47.0	1.0	0.320	63.6	1.0	1.00	54.5	1.0	0.566	48.5	1.0	0.776
Own job/employment	47	72.7	1.0		57.6	1.5		63.6	1.0		60.6	1.3		51.5	1.3	
**Economic hardship**																
No	48	72.9	1.0	0.684	47.1	1.0	0.088	60.4	1.0	0.518	50.0	1.0	0.201	45.8	1.0	0.480
Yes	51	76.5	1.2		58.8	2.0		66.7	1.3		62.7	1.7		52.9	1.7	
**Education**																
≥ High school degree	51	76.5	1.2	0.684	56.8	1.7	0.192	70.6	1.9	0.138	51.0	1.0	0.248	52.9	1.6	0.480
< High school degree	48	72.9	1.0		43.8	1.0		56.3	1.0		62.5	1.6		45.8	1.0	
**Prior housing situation**																
Homeless or precariously housed	55	72.7	1.0	0.605	41.8	1.0	0.053	58.2	1.0	0.207	47.2	1.0	**0.037**	40.0	1.0	**0.035**
Own house or apartment	44	77.3	1.3		61.3	2.2		70.5	1.7		68.2	2.4		61.4	2.4	
**Drug of choice**																
Legal	35	68.6	1.0	0.313	37.1	1.0	0.055	60.0	1.0	0.669	51.4	1.0	0.363	31.4	1.0	**0.009**
Illegal	59	78.0	1.6		57.6	2.3		64.4	1.2		61.0	1.5		59.3	3.2	
**Any mental distress**																
No	49	71.4	1.0	0.452	38.8	1.0	**0.021**	57.1	1.0	0.184	51.0	1.0	0.270	36.7	1.0	**0.012**
Yes	50	78.0	1.4		62.0	2.6		70.0	1.8		62.0	1.6		62.0	2.8	
**Mental distress and SUD**																
No	53	73.6	1.0	0.775	43.4	1.0	0.129	60.4	1.0	0.469	52.8	1.0	0.421	41.5	1.0	0.088
Yes	46	76.1	1.1		58.7	1.9		67.4	1.4		60.8	1.4		58.7	1.4	

*p* < 0.05 are **bolded;** OR = odds ratio; ^a^ number of women in each stratum. ^b^ Prevalence of total IPV (IPV-T) within each stratum (cut off 3). ^c^ Prevalence of severe combined IPV (IPV-SC) within each stratum (cut off 1). ^d^ Prevalence of emotional IPV (IPV-E) within each stratum (cut off 3). ^e^ Prevalence of physical IPV (IPV-P) within each stratum (cut off 2). ^f^ Prevalence of harassment IPV (IPV-H) within each stratum (cut off 2). ^g^ Self-identified; 1 African American woman excluded from the race/ethnicity analysis due to insufficient cell.

**Table 4:** Logistic regression models of IPV by type.

Factor	IPV-T ^a^ OR *	*p*	IPV-SC ^b^ OR *	*p*	IPV-P ^c^ OR *	*p*	IPV-H ^d^ OR *	*p*
**Age, years**								
40+	1.0	0.089	1.0	**0.020**	1.0	**0.039**	1.0	**0.001**
30–39	3.1		4.5		1.5		3.7	
20–29	2.6		3.8		3.1		7.4	
**Child abuse**								
No	1.0	0.111						
Yes	2.0							
**Prior housing situation**								
Homeless or precariously housed			1.0	0.067	1.0	**0.035**	1.0	**0.041**
Own house or apartment			2.2		2.5		2.6	
**Any mental distress**								
No			1.0	**0.025**			1.0	**0.013**
Yes			2.6				3.1	

*p* < 0.05 are **bolded**; * OR = adjusted odds ratio. ^a^ Prevalence of total IPV (IPV-T) within each stratum (cut off 3). ^b^ Prevalence of severe combined IPV (IPV-SC) within each stratum (cut off 1). ^c^ Prevalence of physical IPV (IPV-P) within each stratum (cut off 2). ^d^ Prevalence of harassment IPV (IPV-H) within each stratum (cut off 2).

**Table 5:** Mixed-methods results of analysis of IPV and mental health and substance use, socioeconomic inequality, and racial/ethnic discrimination.

Approach	Quantitative	Qualitative	Convergence, Expansion, and/or Complementarity
**Answer**	**(a) Childhood abuse:**		
	IPV-T was significantly associated with the prevalence of childhood abuse in the univariate model.	Women linked childhood abuse to insufficient family support and resources in the community, e.g., counseling services. They connected abuse to life possibilities, including escaping abusive partners and using substances.	Convergence of both data sets demonstrated a strong and frequent association between abuse during childhood and IPV in adulthood. Qualitative data provided complementarity by suggesting that lack of family support and community resources constrained women’s perceived and actual access to help, and increased their vulnerability to additional harms.
	**(b) Mental health and substance use:**		
	IPV-SC and IPV-H were significantly associated with the prevalence of mental distress.	Fear and anxiety from IPV affected women’s mental health. Many women used alcohol and drugs to cope with IPV. Substance use and fear concerning safety also played roles in decisions to remain with abusive partners.	Convergence of both data sets showed that mental health distress intersected with IPV. Qualitative data provided complementarity by revealing that the relationship was bidirectional. IPV exacerbated mental health and substance use problems. Poor mental health status and substance use may also prevent women from leaving abusive relationships.
	**(c) Socioeconomic conditions:**		
	IPV-P, IPV-SC, and IPV-H were significantly associated with having housing prior to being incarcerated.	Lack of alternative housing, childcare, and finances impacted women’s decisions to remain or return to abusive relationships. Abusive relationships were portrayed as barriers to education and employment.	Convergence of both data sets showed that sparse housing options intersected with IPV. The qualitative data provided expansion regarding the quantitative association between housing and IPV as women who had resided with abusive partners relayed that alternative options for housing, income, and childcare were sparse. This impacted women’s decisions to remain in abusive relationships.
	**(d) Race and ethnicity:**		
	IPV was not significantly associated with race/ethnicity.	Racism pervaded institutions and interactions, adversely affecting women’s mental health and life opportunities.	No convergence was found among data sets, yet qualitative data provided the expansion of quantitative data by revealing that racism compounded socioeconomic marginalization and affected mental health.

## Data Availability

The data presented in this study are available on reasonable request from the corresponding author.

## References

[1] McLeroy K.R., Bibeau D., Steckler A., Glanz K. (1988). An Ecological Perspective on Health Promotion Programs. Health Educ. Q..

[2] Willging C.E., Lilliott E., Kellett N.C., Dlugacz H.A. (2015). Gendered Ideologies of Community Reentry: Contrasting the Perspectives of Rural Women Prisoners, Parole Officers, and Mental Healthcare Personnel. Reentry Planning for Offenders with Mental Disorders, Vol. 2.

[3] Willging C.E., Nicdao E.G., Trott E.M., Kellett N.C. (2015). Structural Inequality and Social Support for Women Prisoners Released to Rural Communities. Women Crim. Justice.

[4] Hegarty K., Bush R., Sheehan M. (2005). The Composite Abuse Scale: Further Development and Assessment of Reliability and Validity of a Multidimensional Partner Abuse Measure in Clinical Settings. Violence Vict..

[5] Karlsson M.E., Zielinski M.J. (2020). Sexual Victimization and Mental Illness Prevalence Rates Among Incarcerated Women: A Literature Review. Trauma Violence Abus..

[6] Khalifeh H., Oram S., Trevillion K., Johnson S., Howard L.M. (2015). Recent intimate partner violence among people with chronic mental illness: Findings from a national cross-sectional survey. Br. J. Psychiatry.

[7] Salom C.L., Williams G.M., Najman J.M., Alati R. (2015). Substance use and mental health disorders are linked to different forms of intimate partner violence victimisation. Drug Alcohol Depend..

[8] Edwards K.M. (2015). Intimate Partner Violence and the Rural–Urban–Suburban Divide: Myth or Reality? A Critical Review of the Literature. Trauma Violence Abus..

[9] Rennison C.M., De Keseredy W.S., Dragiewicz M. (2013). Intimate Relationship Status Variations in Violence Against Women: Urban, Suburban, and Rural Differences. Violence Women.

[10] Hough R.L., Willging C.E., Altschul D., Adelsheim S. (2011). Workforce capacity for reducing rural disparities in public mental health services for adults with severe mental illness. J. Rural. Ment. Health.

[11] Beichner D., Rabe-Hemp C. (2014). “I Don’t Want to Go Back to That Town:” Incarcerated Mothers and Their Return Home to Rural Communities. Crit. Criminol..

[12] Donnermeyer J.F., De Keseredy W. (2014). Rural Criminology: New Directions in Critical Criminology.

[13] Peek-Asa C., Wallis A., Harland K., Beyer K., Dickey P., Saftlas A. (2011). Rural Disparity in Domestic Violence Prevalence and Access to Resources. J. Women’s Health.

[14] Staton M., Ciciurkaite G., Oser C., Tillson M., Leukefeld C., Webster J.M., Havens J.R. (2017). Drug Use and Incarceration among Rural Appalachian Women: Findings from a Jail Sample. Subst. Use Misuse.

[15] Mulder P.L., Lambert W., Coward R.T., Davis L.A., Gold C.H., Smicklas-Wright H., Thorndyke L.E., Vondracek F.W. (2006). Behavioral Health of Rural Women: Challenges and Stressors. Rural Women’s Health: Mental, Behavioral, Physical Issues.

[16] Staton-Tindall M., Duvall J.L., Leukefeld C., Oser C.B. (2007). Health, Mental Health, Substance use, and Service Utilization among Rural and Urban Incarcerated Women. Women’s Health Issues.

[17] Riddell T., Ford-Gilboe M., Leipert B. (2009). Strategies Used by Rural Women to Stop, Avoid, or Escape from Intimate Partner Violence. Health Care Women Int..

[18] Benedini K.M., Fagan A.A. (2020). From Child Maltreatment to Adolescent Substance Use: Different Pathways for Males and Females?. Fem. Criminol..

[19] Daly K. (1992). Women’s Pathways to Felony Court: Feminist Theories of Lawbreaking and Problems of Representation. South. Calif. Rev. Law Womens Stud..

[20] Walters G.D. (2020). Beyond Peer Influence and Selection: A Gendered Pathways Analysis of Social Environmental Predictors of Change in Child and Peer Delinquency. Fem. Criminol..

[21] Reisig M.D., Holtfreter K., Morash M. (2006). Assessing Recidivism Risk Across Female Pathways to Crime. Justice Q..

[22] Kellett N.C., Willging C.E. (2011). Pedagogy of individual choice and female inmate reentry in the U.S. Southwest. Int. J. Law Psychiatry.

[23] Willging C.E., Malcoe L.H., Cyr S.S., Zywiak W.H., Lapham S.C. (2013). Behavioral Health and Social Correlates of Reincarceration Among Hispanic, Native American, and White Rural Women. Psychiatr. Serv..

[24] De Keseredy W., Schwartz M. (2009). Dangerous Exits: Escaping Abusive Relationships in Rural America.

[25] Hunnicutt G. (2009). Varieties of Patriarchy and Violence Against Women. Violence Women.

[26] Oetzel J. (2004). Intimate Partner Violence in American Indian and/or Alaska Native Communities: A Social Ecological Framework of Determinants and Interventions. Am. Indian Alsk. Nativ. Ment. Health Res..

[27] Smith A. (2015). Conquest: Sexual Violence and American Indian Genocide.

[28] Weaver H.N. (2008). The Colonial Context of Violence: Reflections on Violence in the Lives of Native American Women. J. Interpers. Violence.

[29] Friestad C., Åse-Bente R., Kjelsberg E. (2012). Adverse childhood experiences among women prisoners: Relationships to suicide attempts and drug abuse. Int. J. Soc. Psychiatry.

[30] Kelly P.J., Cheng A.-L., Spencer-Carver E., Ramaswamy M. (2014). A syndemic model of women incarcerated in community jails. Public Health Nurs..

[31] Weeks L.E., MacQuarrie C., Begley L., Gill C., Leblanc K.D. (2016). Strengthening resources for midlife and older rural women who experience intimate partner violence. J. Women Aging.

[32] Pedersen J.S., Malcoe L.H., Pulkingham J. (2013). Explaining Aboriginal/Non-Aboriginal Inequalities in Postseparation Violence Against Canadian Women. Violence Women.

[33] Palinkas L.A., Aarons G.A., Horwitz S., Chamberlain P., Hurlburt M., Landsverk J. (2010). Mixed Method Designs in Implementation Research. Adm. Policy Ment. Health Ment. Health Serv. Res..

[34] U.S. Census Bureau (2019). State and County Quick Facts—New Mexico.

[35] Health Resources and Services Administration (2013). Rural Health Information Hub.

[36] McNichol E., Hall D., Cooper D., Palacios V. (2012). Pulling Apart: A State-by-State Analysis of Income Trends.

[37] Rupasingha A., Patrick J.M. (2010). Rural New Mexico Economic Conditions and Trends.

[38] Health Resources and Services Administration (2016). Health Professional Shortage Areas.

[39] New Mexico Department of Health (2014). Health Equity in New Mexico: A Report on Racial and Ethnic Health Disparities.

[40] Institute of Behavioral Research (2007). TCU Drug Screen II (TCUDS II).

[41] Sheehan D., Lecrubier Y., Sheehan K.H., Janavs J., Weiller E., Keskiner A., Schinka J., Knapp E., Sheehan M., Dunbar G. (1997). The validity of the Mini International Neuropsychiatric Interview (MINI) according to the SCID-P and its reliability. Eur. Psychiatry.

[42] American Psychiatric Association (2006). Diagnostic and Statistical Manual of Mental Disorders.

[43] Green B.L., Krupnick J.L., Rowland J.H., Epstein S.A., Stockton P., Spertus I., Stern N. (2000). Trauma History as a Predictor of Psychologic Symptoms in Women with Breast Cancer. J. Clin. Oncol..

[44] Hegarty K., Valpied J. (2007). Composite Abuse Scale Manual.

[45] IBM Corporation (2010). IBM SPSS Statistics for Windows, Version 19.0.

[46] Abramsky T., Watts C.H., Garcia-Moreno C., Devries K., Kiss L., Ellsberg M., Jansen H.A., Heise L. (2011). What factors are associated with recent intimate partner violence? findings from the WHO multi-country study on women’s health and domestic violence. BMC Public Health.

[47] QSR International (2012). NVivo Qualitative Data Analysis Softward.

[48] Patton M.Q. (2015). Qualitative Research & Evaluation Methods: Integrating Theory and Methods.

[49] Corbin J., Strauss A.L. (2008). Basics of Qualitative Research: Techniques and Procedures for Developing Grounded Theory.

[50] Glaser B.G., Strauss A.L. (1967). The Discovery of Grounded Theory: Strategies for Qualitative Research.

[51] Coker D., Macquoid A.D. (2015). Why Opposing Hyper-Incarceration Should Be Central to the Work of the Anti-Domestic Violence Movement. Univ. Miami Race Soc. Justice Law Rev..

[52] Lyon D., Parker B. (2003). Gender-related concerns of rural women with severe and persistent mental illnesses. Arch. Psychiatr. Nurs..

[53] Aday R.H., Dye M.H., Kaiser A.K. (2014). Examining the Traumatic Effects of Sexual Victimization on the Health of Incarcerated Women. Women Crim. Justice.

[54] Widom C.S., Czaja S., Dutton M.A. (2014). Child abuse and neglect and intimate partner violence victimization and perpetration: A prospective investigation. Child. Abus. Negl..

[55] Taft A.J., Hegarty K.L. (2010). Intimate partner violence against women: What outcomes are meaningful?. JAMA.

[56] Asberg K., Renk K. (2015). Safer in Jail? A Comparison of Victimization History and Psychological Adjustment Between Previously Homeless and Non-Homeless Incarcerated Women. Fem. Criminol..

[57] Sikich K.W. (2008). Global Female Homelessness: A Multi-Faceted Problem. Fem. Issues.

[58] Clough A., Draughon J.E., Njie-Carr V., Rollins C., Glass N. (2014). ‘Having housing made everything else possible’: Affordable, safe and stable housing for women survivors of violence. Qual. Soc. Work. Res. Pract..

[59] Logan T., Evans L., Stevenson E., Jordan C.E. (2005). Barriers to Services for Rural and Urban Survivors of Rape. J. Interpers. Violence.

[60] Lanier C., Maume M.O. (2009). Intimate Partner Violence and Social Isolation Across the Rural/Urban Divide. Violence Women.

[61] Carter R.T., Johnson V.E., Kirkinis K., Roberson K., Muchow C., Galgay C. (2019). A Meta-Analytic Review of Racial Discrimination: Relationships to Health and Culture. Race Soc. Probl..

[62] Armstrong K., Putt M., Halbert C.H., Grande D., Schwartz J.S., Liao K., Marcus N., Demeter M.B., Shea J.A. (2013). Prior Experiences of Racial Discrimination and Racial Differences in Health Care System Distrust. Med. Care.

[63] Farmer P. (2004). An Anthropology of Structural Violence. Curr. Anthr..

[64] Galtung J. (1993). Violence Typology: Direct Structural and Cultural Violence. Kult. Gewalt Burger Im Staat.

[65] Farmer P.E., Nizeye B., Stulac S., Keshavjee S. (2006). Structural Violence and Clinical Medicine. PLoS Med..

[66] Garcia A. (2010). The Pastoral Clinic: Addiction and Dispossession along the Rio Grande.

[67] Trujillo M.L. (2009). Land of Disenchantment: Latina/o Identities and Transformations in Northern New Mexico.

[68] Willging C.E., Jaramillo E.T., Mulligan J.M., Castaneda H. (2018). Outsourcing Responsibility: State Stewardship of Behavioral Health Care Services. Unequal Coverage: The Experience of Health Care Reform in the United States.

[69] Metzl J.M., Hansen H. (2014). Structural competency: Theorizing a new medical engagement with stigma and inequality. Soc. Sci. Med..

[70] Herman D., Conover S., Felix A., Nakagawa A., Mills D. (2007). Critical Time Intervention: An Empirically Supported Model for Preventing Homelessness in High Risk Groups. J. Prim. Prev..

[71] Messina N.P., Braithwaite J., Calhoun S., Kubiak S. (2016). Examination of a Violence Prevention Program for Female Offenders. Violence Gend..

[72] Kessler R., Stafford D. (2008). Primary Care Is the De Facto Mental Health System. Collaborative Medicine Case Studies.

[73] Padgett D.K., Henwood B.F., Tsemberis S. (2016). Response to Review of Housing First: Ending Homelessness, Transforming Systems, and Changing Lives. Psychiatr. Serv..

[74] The National Domestic Violence Hotline Create a Safety Plan, n.d. https://www.thehotline.org/plan-for-safety/create-a-safety-plan/.

[75] Wattanaporn K.A., Holtfreter K. (2014). The Impact of Feminist Pathways Research on Gender-Responsive Policy and Practice. Fem. Criminol..

[76] Jones N.L., Breen N., Das R., Farhat T., Palmer R. (2019). Cross-Cutting Themes to Advance the Science of Minority Health and Health Disparities. Am. J. Public Health.

[77] Smith A., Richie B.E., Sudbury J. (2006). The Color of Violence: INCITE! Anthology.

[78] Collins J.L., Barboa A. (2017). A Path Forward: Ending Gender-Based Violence in New Mexico.

[79] Travis J., Western B., Redburn S. (2014). The Growth of Incarceration in the United States: Exploring Causes and Consequences.

[80] Lyon E. Under “In-House Parole,” New Mexico Prisoners Remain in Prison. Prison Legal News 2018. https://www.prisonlegalnews.org/news/2018/dec/5/under-house-parole-new-mexico-prisoners-remain-prison/.

